# Sanitary Department of the Journal

**Published:** 1881-11

**Authors:** 


### SANITARY DEPARTMENT OF "HALL'S JOURNAL OF HEALTH."

We are daily receiving requests from our readers to purchase for them "HEALTH FOODS," "FOOD MEDICINES," PURE BAKING POWDERS, and various MEDICINES that our experience has taught us the value of ; also standard books ; and last but not least, many families rely on us to procure for them PURE BOVINE VACCINE MATTER, which is the only vaccine matter that is always safe. These commissions already keep one person employed much of the time. We have therefore determined to make this a branch of our regular business, and announce ourselves ready to fill orders for GLUTEN OF WHEAT, GLUTEN COFFEE, COOKED FOODS, as CRUSHED WHEAT, BARLEY, OATS, CORN and RYE, also the various preparations of MALT, and for books, medicines, vaccine matter and any other like articles that can be found in New York market.

E. H. GIBBS & CO.

---

AMONG many curious discoveries recently made by the Paris bookworms, among the works of the early Church Fathers, the most notable are the two following : St. Clement, of Alexandria, thus sketches the Saviour : "Jesus had no beauty of face ; his person offered no physical attractions ; he only possessed beauty of soul, which is true beauty." St. Irenæus, a disciple of St. Polycarp, who was a disciple of St. John, wrote that his master had often heard the beloved disciple say that the hair of Jesus had already turned white when he began his mission. If this latter be true, it is a fact full of deep significance and of most pathetic interest.

---

SAID Mrs. Younghusband, "Charley, why is it you never talk with me as you did before we were married? I notice that you talk fast enough with other women." "Dearest," replied Charley, without taking his eyes off his newspaper, "you know that people talk to conceal their thoughts ? I have nothing to conceal from you, love." In another moment he was deep in the stock market reports, while something that sounded very much like "humbug" trembled on the lips of Mrs. Younghusband as she slowly left the room.—*Boston Transcript*..

---

VENTURE not into the company of those that are infected with the plague ; no, though thou think thyself guarded with an antidote.

### LIFE INSURANCE WITHIN THE REACH OF ALL.

INSURING one's life, which most people deem a duty, has hitherto been a luxury too costly for people of moderate means. Life insurance companies, in order to accumulate their enormous surplus funds, in many cases amounting to millions of dollars, have fixed their rates of premiums at figures which, we repeat, are prohibitory.

The extravagance of what we may properly call this old system of insurance has driven great numbers of persons to the adoption of a newer and better system of life insurance, within the reach of those to whom it is of the greatest use, to wit, persons of moderate means. This system is strictly mutual, and therefore the safest of any ; since, after providing for the ordinary expenses of economical management, members are only taxed the small sums required from time to time to make up the losses by death ; thus members are not taxed three or four times as much as is necessary in order to establish an enormous surplus fund in which they have no interest beyond the amount of their insurance. We believe in the new plan of life insurance, and have shown our faith by taking policies of insurance in two associations. We will forward all necessary printed information to any reader of the JOURNAL who will take the trouble to write to us on the subject.

